# Colloidal Characteristics of Poly(L-Lactic Acid)-b-Poly (ε-Caprolactone) Block Copolymer-Based Nanoparticles Obtained by an Emulsification/Evaporation Method

**DOI:** 10.3390/polym16192748

**Published:** 2024-09-28

**Authors:** Oana Cucoveica, Carmen Stadoleanu, Christelle Bertsch, Romain Triaud, Iustina Petra Condriuc, Leonard Ionut Atanase, Christelle Delaite

**Affiliations:** 1 “Cristofor Simionescu” Faculty of Chemical Engineering and Environmental Protection, “Gheorghe Asachi” Technical University of Iasi, 700050 Iasi, Romania; oanacucoveica@yahoo.com; 2 Faculty of Medicine, Apollonia University of Iasi, 700511 Iasi, Romania; drcarsta@yahoo.com; 3 Laboratoire de Photochimie et d’Ingénierie Macromoléculaires (LPIM), Université de Haute Alsace (UHA), 68100 Mulhouse, France; c.bertsch@unistra.fr (C.B.); triaud.romain@gmail.com (R.T.); 4 Université de Strasbourg, 67000 Strasbourg, France; 5 Faculty of Medicine, Grigore T. Popa University of Medicine and Pharmacy, 700115 Iasi, Romania; iustina_condriuc@yahoo.com; 6 Academy of Romanian Scientists, 050045 Bucharest, Romania

**Keywords:** poly(lactic acid), poly(ε-caprolactone), polyester, block copolymers, nanoparticles, emulsion/evaporation method

## Abstract

Poly(L-lactic acid) (PLLA) and poly(ε-caprolactone) (PCL), two biodegradable and biocompatible polymers that are commonly used for biomedical applications, are, respectively, the result of the ring-opening polymerization of LA and ε-CL, cyclic esters, which can be produced according to several mechanisms (cationic, monomer-activated cationic, anionic, and coordination-insertion), except for L-lactide, which is polymerized only by anionic, cationic, or coordination-insertion polymerization. A series of well-defined PLLA-b-PCL block copolymers have been obtained starting from the same PLLA homopolymer, having a molar mass of 2500 g·mol^−1^, and being synthesized by coordination-insertion in the presence of tin octoate. PCL blocks were obtained via a cationic-activated monomer mechanism to limit transesterification reactions, and their molar masses varied from 1800 to 18,500 g·mol^−1^. The physicochemical properties of the copolymers were determined by ^1^H NMR, SEC, and DSC. Moreover, a series of nanoparticles (NPs) were prepared starting from these polyester-based copolymers by an emulsification/evaporation method. The sizes of the obtained NPs varied between 140 and 150 nm, as a function of the molar mass of the copolymers. Monomodal distribution curves with PDI values under 0.1 were obtained by Dynamic Light Scattering (DLS) and their spherical shape was confirmed by TEM. The increase in the temperature from 25 to 37 °C induced only a very slight decrease in the NP sizes. The results obtained in this preliminary study indicate that NPs have a temperature stability, allowing us to consider their use as drug-loaded nanocarriers for biomedical applications.

## 1. Introduction

Nanomaterials and nanotechnologies, used in general for therapeutic purposes, are a fast-growing area of research due to the need to develop new therapeutic systems. Today, the delivery of therapeutic molecules to diseased organs, tissues, or cells is a significant challenge in the treatment of human diseases, particularly cancer. Adapted to current medical and societal challenges, nanotechnologies for therapeutics and/or diagnostics represent a high-potential sector for implementing such treatments, thanks to the concept of drug vectorization [1,2,3,4].

Drug delivery and the controlled delivery of bioactive compounds bring with them constraints in terms of toxicity, (bio)degradability, and biocompatibility. Due to strict constraints, a limited number of polymers can be used as drug delivery systems (DDSs). Among them, polyesters are widely developed today because of their very low toxicity, biocompatibility, and ability to degrade by chemical or enzymatic hydrolysis into residues that the body can assimilate. Among them, poly(lactide) (PLA) and poly(ε-caprolactone) (PCL) are of particular interest [5,6,7].

Polylactide (PLLA) is not only biodegradable but also bio-resorbable, since its hydrolysis in physiological media gives non-toxic components that are eliminated via the Krebs cycle as water and carbon dioxide [8]. Poly(*ε*-caprolactone) (PCL) is a slow-degrading aliphatic polyester compared to PLA; therefore, it is most suitable for the design of long-term, implantable drug-delivery systems. Being FDA-approved, it is a widely utilized polymer for biomedical applications, currently regarded as a non-toxic and tissue-compatible material [9,10].

These polyesters can be synthesized in different ways, but the most prevalent is the ring-opening polymerization (ROP) of cyclic esters. Among the various ROP processes, including anionic, cationic, coordination–insertion, and cationic-activated monomer polymerizations, coordination-insertion has gained increasing attention for the polymerization of ε-caprolactone and lactide [11,12,13,14].

PLLA is brittle due to its high glass transition temperature, while PCL has a glass transition temperature well below room temperature; thus, it behaves as a tough polymer [15]. Copolymers and blends of PLLA and PCL have been developed in order to take advantage of the improved synergic properties offered by both homopolymers. Copolymerization represents a better route than blending due to the immiscibility of homopolymers. In addition, copolymerization provides a means to adjust the degradation rate, as well as the physical and mechanical properties. Feng et al. [16] and Ye et al. [17] reported, for example, the synthesis of block copolymers based on DL-lactide and ε-caprolactone to take advantage of the degradability of PLLA and the permeability to drugs of PCL. Investigations were focused on the effects of composition, molar mass, and drug loading on the degradability and the permeability of the block copolymers [18].

The advantages of polyesters are numerous: (i) the possible hydrolytic or enzymatic hydrolysis in a biological environment of ester bonds; (ii) a degradation rate adjustable by the nature of the monomers and their ratio, as well as by the length of the chains; (iii) simple and reproducible synthesis methods; (iv) generally non-toxic degradation products that can be eliminated by normal metabolic pathways [19,20]. 

In the biomedical field, the challenges related to drug administration are numerous: solubility in aqueous environment, stability issues in biological environments, limitations in transport and biodistribution, and problems related to the low therapeutic concentration. To overcome these drawbacks, different controlled drug delivery systems have been studied in order to ensure that the drug releases at the proper moment in a secure and reproducible manner, continuously, and at a concentration that is within the therapeutic limits.

Among the numerous existing drug nanocarriers, polymeric nanoparticles (NPs) stand out due to their stability and biocompatibility [21]. Moreover, the NPs obtained from polyester-based copolymers are known to be more biocompatible and biodegradable than many other carriers, which explains their popularity for the development of DDSs. The stability of these systems is appreciable with regard to the requirements of long blood circulation time in order to take advantage of the permeability and retention effect. Another important advantage of polyester-based NPs is the possibility of modulating the release of the active principle as a function of the polymer matrix molecular characteristics [22]. The preparation method of NPs plays a fundamental role in their physicochemical and biological properties. These types of polyester-based NPs, with sizes between 50 and 200 nm, can be obtained by different methods, such as nanoprecipitation, emulsion/solvent evaporation, and emulsion/solvent diffusion [23].

In the present study, polyester-based NPs were obtained by an emulsion/evaporation solvent method, which consisted of dissolving the copolymer and/or a hydrophobic molecule in a common and volatile solvent that was immiscible with water. An intense energy input is necessary to obtain an emulsion, generally through very high-speed homogenization or by sonication. A surfactant previously incorporated into the aqueous phase is necessary to maintain the emulsion stability. Once the emulsion is obtained, the solvent is eliminated, most often by evaporation. The size and size distribution of the obtained NPs are dependent on different factors, such as the nature of the organic solvent, copolymer concentration in the organic phase, organic solvent/water ratio, emulsification duration and rate, and the nature and concentration of surfactant [23,24,25].

The main objective of this study was to prepare NPs, by the emulsification/evaporation method, starting from original PLLA-b-PCL block copolymers with different PCL lengths in order to investigate the influence of the molecular characteristics of copolymers on the colloidal characteristics of the free and drug-loaded particles. The block copolymers have been obtained from the same PLLA homopolymer, synthesized by coordination-insertion in the presence of tin octoate. In contrast, the PCL blocks were obtained via a cationic-activated monomer mechanism, in order to limit transesterification reactions. Several physicochemical methods—NMR, SEC and DSC—were used for the characterization of these polyester-based copolymers in order to assess their molecular characteristics. The obtained NPs were analyzed by Dynamic Light Scattering (DLS) at 25 and 37 °C.

## 2. Materials and Methods

### 2.1. Materials

ε-caprolactone (ε-CL) (purity 99%), benzyl alcohol (99% purity), and toluene (purity ≥ 99.3%) purchased from Alfa Aesar (Haverhill, MA, USA) were dried and stored on a 3 Å molecular sieve under nitrogen. L-lactide (LA) (97% purity), purchased from Corbion (Amsterdam, Netherlands), was stored in a desiccator, under vacuum, and oven-dried (60 °C) at least 24 h before each use. Tin 2-ethylhexanoate (tin octoate, Sn(Oct)_2_, purity 92.5–100%), purchased from Sigma Aldrich (St. Louis, MO, USA), was used as received. Para-toluenesulfonic acid monohydrate (PTSA) from Sigma-Aldrich (St. Louis, MO, USA) (purity ≥ 98.5%) was stored in a drawer, under vacuum, to protect it from moisture. Poly(vinyl alcohol) (PVA) with M_w_ = 13,000–23,000 g·mol^−1^, hydrolysis degree of 87–89%, was purchased from Carlo Erba (Emmendingen, Germany).

### 2.2. Synthesis of Block Copolymers

Polymerizations were carried out in a Schlenk flask (250 mL) conditioned by 3 “vacuum-nitrogen” cycles in order to proceed under an inert and anhydrous atmosphere. All the glassware used was stored in an oven (70 °C) before use. The poly(L-lactide) was synthetized by ring-opening polymerization (ROP) in bulk, by adding the initiator (benzyl alcohol), the catalyst (Sn(Oct)_2_, 10 molar % compared to benzyl alcohol), and the L-lactide in the flask, under nitrogen flow, to synthesize the first block of the copolymer. The temperature was raised to 150–160 °C for 18 h to reach maximum conversion. At the end of the LA polymerization, the PLLA obtained was purified by dissolution in toluene and precipitation in cold ethanol and dried under vacuum. Then, the PCL block was synthesized in toluene, via cationic monomer-activated ROP, in order to limit transfer reactions, using paratoluene sulfonic acid (PTSA) as a catalyst. Necessary amounts of PLLA and ε-CL, depending on the targeted molar masses of PCL, were incorporated into a Schlenk flask heated to 85 °C during x hours, depending on the targeted PCL molar mass. The amount of PTSA was chosen to have a PLLA/catalyst molar ratio of 10%. All copolymers were then recovered by precipitation in cold ethanol, followed by vacuum drying to remove toluene and possible traces of monomer. The physicochemical properties were determined by ^1^H NMR, SEC, and DSC. A typical example of a synthesis of PLLA-b-PCL diblock copolymer (DB3 sample) proceeds as follows. L-lactic acid (60 g), benzyl alcohol (2.10 mL), and tin octoate (0.65 mL) were introduced into a Schlenk flask for the synthesis of the PLLA precursor in bulk (M_n_ = 2500 g·mol^−1^ determined by ^1^H NMR). Then, 9.80 g of purified PLLA, 15.55 mL of ε-CL, 100 mL of toluene, and 27.6 mg of PTSA were introduced into a Schlenk flask. The polymerization was carried out under nitrogen at 85 °C for 40 h. At the end of the synthesis, the resulting crude product was precipitated into cold ethanol (300 mL) to remove unreacted monomers.

### 2.3. Preparation of NPs by an Emulsification/Evaporation Method

In a typical procedure, a series of NPs were obtained, starting from 5 mL of copolymer solution in chloroform at 1 wt.%. After the complete dissolution of the copolymer, the solution was added drop by drop under vigorous stirring into 60 mL of aqueous PVA solution at a concentration of 1 wt.%. The emulsion thus obtained is left under magnetic stirring at 500 rpm for 24 h at room temperature for the evaporation of the organic solvent. After this period, the dispersion is diluted in water and analyzed by Dynamic Light Scattering (DLS) (3–5 drops of dispersion in 5 mL of water). NPs were recovered as a dry powder by freeze-drying in the presence of trehalose as a cryo-protector, at a concentration of 10 wt.% compared to the copolymer, and kept at 4 °C for further tests. Dry NPs were easily redispersed in water at a concentration of 0.1 wt.% and analyzed by DLS at 25 and 37 °C. Each NP suspension was prepared in triplicate. and average values were calculated.

### 2.4. Characterization Techniques

^1^H Nuclear Magnetic Resonance (NMR) spectra were acquired on a Bruker AC-400F (Berlin, Germany) operating at 400 MHz in CDCl_3_, and spectra were referenced to the residual deuterated solvent peak. Using the peak assignments obtained by ^1^H NMR, it was possible to calculate the mean Polymerization Degree (*DPn*) of *PLLA* and *PCL* from Equations (1) and (2):

(1) $$DPn\left(PLLA\right)=\frac{\delta \left(b,c,c^{\prime }\right)-2}{2}$$

(2) $$DPn\left(PCL\right)=\frac{\delta \left(\varepsilon \right)-\delta \left(\varepsilon^{\prime }\right)}{2}$$

The molar masses of each block were then determined using Equations (3) and (4):

(3) Mn (PLLA)=DPn (PLLA)×144+M (benzyl alcohol)

(4) Mn (PCL)=DPn (PCL)×114

The molar mass of the block copolymer is then calculated according to Equation (5):

(5) Mn (PLLA−b−PCL)=Mn (PLLA)+Mn (PCL)

Molar masses (*Mn* and *Mw*) and dispersity *Mw*/*Mn*, also designated Ð, were determined using size exclusion chromatography (SEC) analysis performed on a Shimadzu LC-20AD (Kyoto, Japan) liquid chromatograph equipped with two Varian PL gel 5 μm MIXED-C columns, 300 mm × 7.5 mm, coupled to a high-resolution refractive index detector (Shimadzu RID-10A) (refractometer temperature: 30 °C; injection volume: 100 μL). THF was used as an eluent at a flow rate of 1.0 mL.min^−1^. Sample concentration was 10 mg.mL^−1^ and the molar masses were determined relative to a polystyrene calibration curve.

Thermal analysis (glass transition temperatures and melting enthalpies) was performed by differential scanning calorimetry (DSC) (TA Instruments Model Q200) (New Castle, DE, US). The samples (5–15 mg in a non-hermetic aluminium capsule) were heated by performing two successive temperature ramps under nitrogen, between −80 °C and +200 °C, at a speed of 10 °C.min^−1^. The measurements were carried out according to the following cycles: 25 °C to +200 °C with a heating rate of 10 °C.min^−1^; +200 °C to −80 °C with a cooling rate of 10 °C.min^−1^; −80 °C to +200 °C with a heating rate of 10 °C.min^−1^; +200 °C to 25 °C with a cooling rate of 20 °C.min^−1^. The crystallinity of the PCL block was assumed to be proportional to the experimental heat of fusion 139.5 J.g^−1^ for the 100% crystalline PCL, according to Crescenzi et al. [26], by taking into account the PCL mass ratio in the block copolymer.

NP size and size distribution were determined by Dynamic Light Scattering (DLS) using a Malvern Zetasizer Pro (Malvern, Worcestershire, UK), equipped with a 4 mW He–Ne laser at a wavelength of 532 nm and at a scattering angle of 173°. Based on the Stokes–Einstein equation, the hydrodynamic diameter (volume average) Dv, the Z-average diameter, which is an intensity-weighted size average, and the polydispersity index (PDI) of the sample are available. The data were collected for 5 consecutive measurements in automatic mode, typically requiring a measurement duration of 70 s. A Philips CM100 microscope, equipped with an Olympus camera, was used for the TEM microscopy. NP aqueous suspensions were displayed on a formvar-coated copper grid and analyzed after water evaporation. 

## 3. Results

### 3.1. Diblock Copolymer Synthesis

**Scheme 1:**
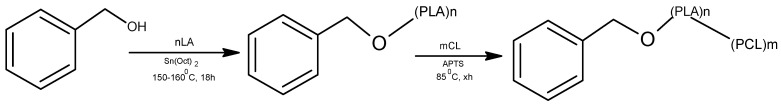
Synthesis of PLLA-b-PCL copolymers.

Syntheses of PLLA-b-PCL diblock copolymers have been carried out in two steps, by ring-opening polymerization, by successively adding L-lactide and ε-caprolactone, as described previously and presented in Scheme 1. This strategy was employed to avoid transesterification reactions observed when Sn(Oct)_2_ was also used to synthesize the second PCL block.

A series of different diblock copolymers, starting from the same PLLA block with increasing lengths of the PCL sequence, were obtained, and the molecular characteristics are provided in Table 1.

**Figure 1:**
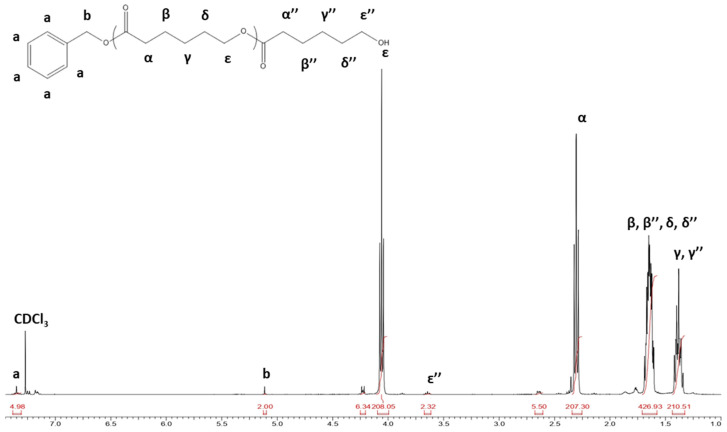
^1^H NMR spectrum of PLLA-b-PCL copolymer (DB_4_) in CDCl_3_.

Figure 1 and Table 2 present the ^1^H NMR spectra and the assignments of a PLLA-b-PCL block copolymer in deuterated chloroform.

Characteristic signals of PLLA and PCL units were observed. Signals at 1.50 ppm (-CH_3_; letter d) and 5.15 ppm (-CH; letter c) were assigned to PLLA repetitive units. Moreover, signals at 1.42 ppm, 1.65 ppm, 2.30 ppm, and 4.05 ppm refer to the different methylene protons (-CH_2_-) of PCL blocks. Therefore, the quasi-absence of signals at 2.40 ppm, 4.15 ppm, and 5.15 ppm, characteristic of LA-CL junctions, show a quasi-absence of intra- and intermolecular transesterification reactions, confirming a well-defined diblock copolymer structure.

**Figure 2:**
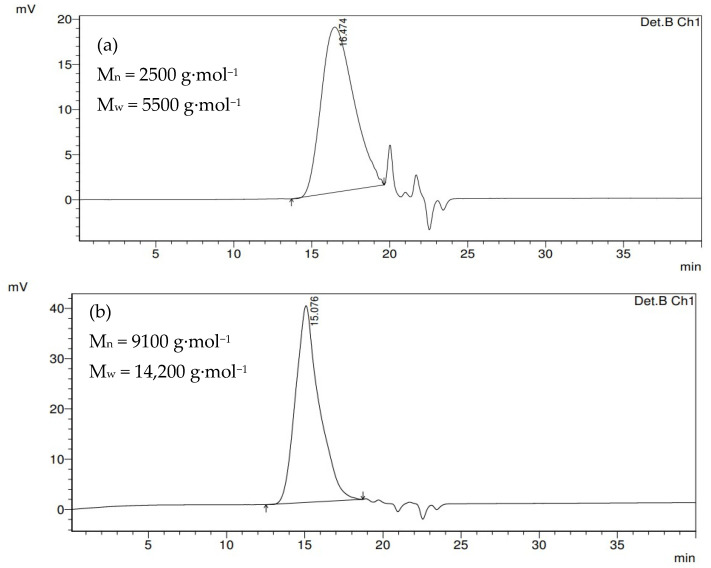
SEC chromatograms of PLLA precursor (**a**) and PLLA-b-PCL copolymer (DB_2_) (**b**).

As an example, the SEC chromatograms of PLLA and a PLLA-b-PCL diblock copolymer (DB_2_ sample) are shown in Figure 2a and Figure 2b, respectively.

The M_n_ of the copolymer is 9100 g·mol^−1^ (according to a PS calibration curve), while the M_n_ of PLLA is 2500 g·mol^−1^. The M_n_ of the copolymer is higher than that of PLLA, which means there was indeed a re-priming of PCL from PLLA. Moreover, the single monomodal peak confirms the presence of one population, and, therefore, the absence of residual PLLA. This result shows that a block copolymer has been obtained and excludes the possibility of having a mixture of two homopolymers.

The dispersity of PLLA synthesized in the presence of Sn(Oct)_2_ is relatively high (Ð = 2) but remains consistent with values found in the literature [27]. This high dispersity value is related to intermolecular transesterification reactions supported by a temperature of 150–160 °C, the melting temperature of PLLA, and by bulk PLLA synthesis. In bulk, there are indeed diffusion problems related to the high viscosity of the medium. However, the dispersity decreases after ε-CL’s polymerization because the cationic polymerization via the activated monomer technique is well-controlled. In addition, the relatively low synthesis temperature limited the transesterification reactions [11].

**Figure 3:**
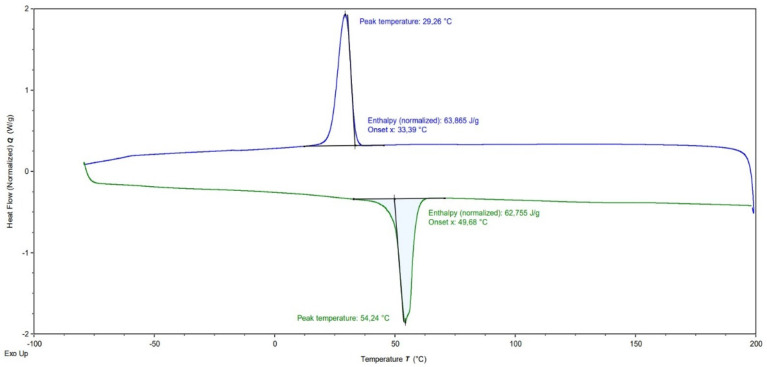
DSC thermogram of DB_5_ sample (cooling ramp and second ramp of heating).

No significant differences were noticed for the thermal behavior of the obtained samples. To illustrate this, Figure 3 shows a double cooling/heating cycle from 250 °C to −80 °C and from −80 °C to 250 °C (second ramp).

The glass transition temperatures (T_g_) of PLLA and PCL are around 60 °C and −60 °C, respectively, according to the literature data [28,29]. However, the DSC analyses do not show a clearly observable T_g_ for PLLA block due to the overlap with the melting peak of PCL, also around 60 °C. The T_g_ of the PCL block cannot be observed on the different thermograms. The DSC analysis of the different PLLA-b-PCL copolymers of varying compositions confirms the block structure of the copolymers since the melting temperature (T_f_) of the main PCL segments is observed around 50–55 °C. On the other hand, it is unsurprising to observe no melting peak around 160–165 °C, corresponding to the central segment of PLLA, its composition being largely lower than that of PCL. Moreover, the values of the PCL crystallinity ratio increase with the PCL length and stabilize around 50% for a length of around 10,000 g/mol, in agreement with the literature data [30].

Table 2 shows that the cationic polymerization of ε-CL from PLLA via monomer-activated cationic technique is a good method to obtain well-defined block copolymers. These results show a good agreement between the theoretical and experimental values of the molar masses, which allows us to validate the implemented protocol. In conclusion, the combination of the coordination-insertion bulk polymerization of L-lactide (in the presence of tin octoate) followed by the cationic polymerization of ε-caprolactone in solution by a monomer-activated cationic technique (in the presence of PTSA) allows us to obtain block copolymers of controlled molar masses and relatively low dispersity.

### 3.2. From Controlled Block Copolymers to Nanoparticles

First of all, it was interesting to investigate the evolution of the NP sizes at each step of the preparation method. In this context, Table 3 provides the sizes of NPs obtained from sample DB_3_ as a function of temperature.

**Figure 4:**
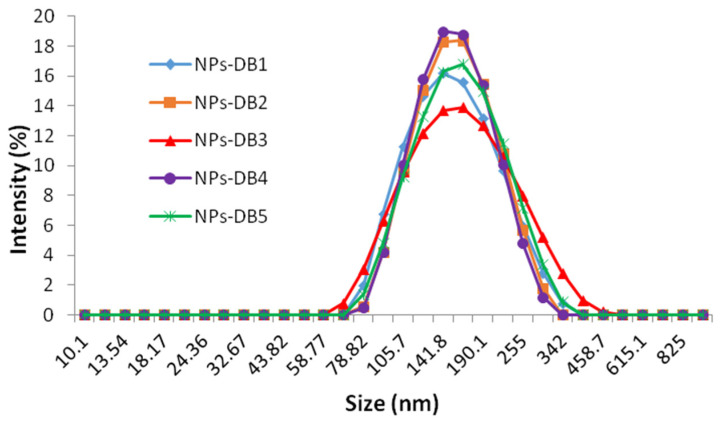
Distribution curves of freeze-dried NPs in the presence of trehalose at 37 °C.

The data provided in Table 3 show a slight agglomeration of the NPs after freeze-drying in the absence of trehalose (higher Z-average and PDI values). However, no agglomeration is noticed after freeze-drying in the presence of trehalose, which indicates the beneficial role of trehalose, even if the NP sizes slightly increase in the presence of cryoprotector. All the NP samples were further analyzed after freeze-drying in the presence of trehalose, and the distribution curves, at 37 °C, are shown in Figure 4.

**Figure 5:**
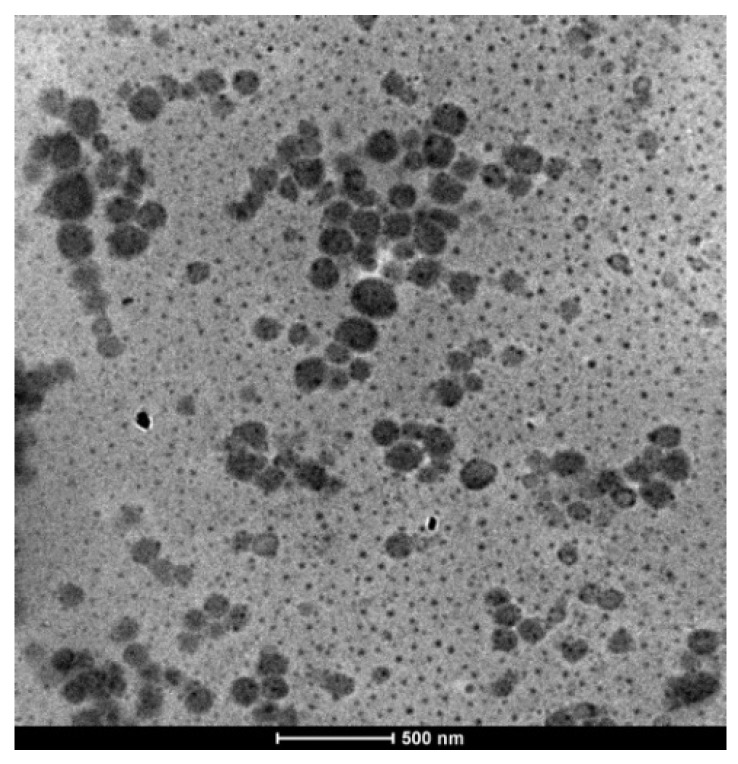
TEM micrograph of DB_1_ sample.

From this figure, it can be noticed that all the curves are monomodal with a very narrow PDI value. The morphology of the NPs was investigated by TEM, and it appeared that they had a spherical shape, while no agglomeration was observed (Figure 5).

The complete colloidal characteristics, obtained by DLS, of the NPs prepared from all the copolymers are presented in Table 4.

From Table 4, it can be observed, as a general remark, that all the NPs have sizes around 150 nm and low PDI values, generally under 0.01, which confirm their very narrow polydispersity. Moreover, it appears that, by increasing the molar mass of the copolymers, from DB_1_ to DB_5_, only a slight increase in the NPs’ sizes is observed, whereas no evident correlation can be noticed for PDI values. Comparing the NP sizes as a function of temperature, it seems that, by increasing the temperature from 25 to 37 °C, a very slight size decrease occurs that is, however, within the experimental error limits. As a function of the molar mass of the copolymers, a similar trend is seen at 37 °C for both Z-average and Dv values. The increase in the NP sizes as a function of the molar mass of the copolymers was also noticed by other authors [31].

Considering the colloidal characteristics of the obtained NPs, it can be stated that these particles can be used as nanocarriers to prepare drug delivery systems with biomedical applications.

## 4. Conclusions

In the present study, the polymerization of L-lactide was performed by coordination-insertion polymerization, while ε-caprolactone was polymerized via the activated monomer cationic technique in order to limit transesterification reactions. By using these distinct polymerization techniques, it was possible to obtain a series of well-defined block copolymers that had a starting block of a PLLA sequence with an M_n_ of 2500 g·mol^−1^ and a second PCL block with increasing M_n_ values from 1800 to 18,500 g·mol^−1^, in order to modify the biodegradability rate. These polyester-based copolymers were characterized by correct dispersity values and high purity. Having in mind their molecular characteristics, it is interesting to evaluate the influence of the molar masses on the NPs’ sizes. Spherical NPs with sizes around 150 nm and low PDI values, lower than 0.1, were obtained by an emulsification/evaporation method. It was shown that the increase in the molar mass of the copolymers led to the increase in the average sizes from around 140 to 150 nm, with no influence on the PDI values. Moreover, the increase in the temperature from 25 to 37 °C has a limited effect on the NPs sizes. The results of this preliminary study concerning the colloidal characteristics of the NPs demonstrate that they have suitable sizes and stability for further biomedical applications. In a further study, drug-loaded NPs will be prepared and characterized.

## Figures and Tables

**Table 1:** Molecular characteristics of synthesized diblock copolymers.

Sample	M_n_ PLLA ^b^ (g·mol^−1^)	Theor. DP_n_ PCL	Exp. DP_n_ PCL^a^	M_n_ PCL ^a^ (g·mol^−1^)	M_n_ Cop. ^a^ (g·mol^−1^)	M_n_ Cop. ^b^ (g·mol^−1^)	Ð ^b^	T_m_ (°C) ΔH_m_ (J.g^−1^) ^c^	T_C_ ^c^ (°C)	χ_c_^c^ (%)
DB_1_	2500	18	16	1800	4300	5700	1.45	48.2 17.7	16.0	30.8
DB_2_		53	77	8800	11,300	9100	1.56	52.8 41.8	30.3	38.5
DB_3_		88	85	9800	12,300	10,200	1.46	52.0 57.2	28.0	51.5
DB_4_		123	143	16,300	18,800	12,700	1.41	53.4 63.5	30.3	52.5
DB_5_		175	162	18,500	21,000	13,200	1.41	49.7 62.8	29.3	51.0

^a^ Calculated from ^1^H NMR analysis. ^b^ Values obtained by SEC in THF with respect to polystyrene standards. ^c^ Obtained by DSC (second heating ramp).

**Table 2:** Chemical shifts of protons of the different units present on the copolymers.

Signal Groups	Type of Proton	Assigned Letter	δ (ppm)
Initiator’s groups	**Bn**-CH_2_-O- O-CH_2_-**Bn**-CH_2_-O-	a	7.35
	Bn-**CH_2_**-O- O-**CH_2_**-Bn-**CH_2_**-O-	b	5.10
Repetitive units	-(CO-**CH**(CH_3_)-O)_n_-	c	5.15
	-(CO-CH(**CH_3_**)-O)_n_-	d	1.50
Intermediary groups	-(CO-**CH**(CH_3_)-O)-	c′	5.15
	-(CO-CH(**CH_3_**)-O)-	d′	1.50
	-(CO-**CH_2_**-CH_2_-CH_2_-CH_2_-CH_2_-O)-	α′	2.40
	-(CO-CH_2_-**CH_2_-CH_2_-CH_2_**-CH_2_-O)-	β′, γ′, δ′	1.42/1.65
	-(CO-CH_2_-CH_2_-CH_2_-CH_2_-**CH_2_**-O)-	ε′	4.15
Repetitive units	-(CO-**CH_2_**-CH_2_-CH_2_-CH_2_-CH_2_-O)_n_-	α	2.30
	-(CO-CH_2_-**CH_2_-CH_2_-CH_2_**-CH_2_-O)_n_	β, γ, δ	1.42/1.65
	-(CO-CH_2_-CH_2_-CH_2_-CH_2_-**CH_2_**-O)_n_-	ε	4.05
Terminal groups	-CO-**CH_2_**-CH_2_-CH_2_-CH_2_-CH_2_-OH	α″	N/A
	-CO-CH_2_-**CH_2_-CH_2_-CH_2_**-CH_2_-OH	β″, γ″, δ″	N/A
	-CO-CH_2_-CH_2_-CH_2_-CH_2_-**CH_2_**-OH	ε″	3.65
Residual groups	α-CL	N/A	2.63
	ε-CL	N/A	4.23
	**CH**-LA	N/A	5.03

N/A—non available.

**Table 3:** Z-average and PDI values of NPs obtained from DB_3_ sample after solvent evaporation, before freeze-drying and after freeze-drying, in the presence or absence of trehalose.

Temperature (°C)	Parameters	After Solvent Evaporation	After Addition of Trehalose	After Freeze-Drying in the Absence of Trehalose	After Freeze-Drying in the Presence of Trehalose
25	Z-average (nm)	128.5 ± 0.1	139.2 ± 0.3	173.3 ± 0.8	151.4 ± 0.4
	PDI	0.066 ± 0.01	0.071 ± 0.3	0.134 ± 0.02	0.069 ± 0.01
37	Z-average (nm)	127.8 ± 0.2	138.9 ± 0.2	172.4 ± 0.4	150.3 ± 0.2
	PDI	0.064 ± 0.01	0.078 ± 0.2	0.127 ± 0.01	0.099 ± 0.01

**Table 4:** Z-average, Dv, and PDI values, at 25 and 37 °C, of the NPs at a concentration of 0.1 wt.% in water.

T (°C)	Parameters	NPs-DB_1_	NPs-DB_2_	NPs-DB_3_	NPs-DB_4_	NPs-DB_5_
25	Z-average (nm)	144.3 ± 0.2	148.9 ± 0.3	151.4 ± 0.4	151.7 ± 0.4	153.1 ± 0.3
	Dv (nm)	143.8 ± 0.3	148.2 ± 0.2	150.9 ± 0.3	151.2 ± 0.4	153.0 ± 0.2
	PDI	0.114 ± 0.02	0.078 ± 0.01	0.069 ± 0.01	0.078 ± 0.01	0.088 ± 0.01
37	Z-average (nm)	143.5 ± 0.4	148.0 ± 0.1	150.3 ± 0.2	150.9 ± 0.5	152.8 ± 0.4
	Dv (nm)	143.2 ± 0.3	147.5 ± 0.2	149.5 ± 0.3	150.2 ± 0.3	152.5 ± 0.3
	PDI	0.097 ± 0.01	0.072 ± 0.01	0.099 ± 0.01	0.070 ± 0.01	0.072 ± 0.01

## Data Availability

The original contributions presented in the study are included in the article, further inquiries can be directed to the corresponding author.
